# Glycoengineering Chinese hamster ovary cells: a short history

**DOI:** 10.1042/BST20200840

**Published:** 2021-03-11

**Authors:** Roberto Donini, Stuart M. Haslam, Cleo Kontoravdi

**Affiliations:** 1 Department of Life Sciences, Imperial College London, London SW7 2AZ, U.K.; 2 Department of Chemical Engineering, Imperial College London, London SW7 2AZ, U.K.

**Keywords:** cell engineering, erythropoietin, glycomics, glycoproteomics, glycosylation, monoclonal antibodies

## Abstract

Biotherapeutic glycoproteins have revolutionised the field of pharmaceuticals, with new discoveries and continuous improvements underpinning the rapid growth of this industry. N-glycosylation is a critical quality attribute of biotherapeutic glycoproteins that influences the efficacy, half-life and immunogenicity of these drugs. This review will focus on the advances and future directions of remodelling N-glycosylation in Chinese hamster ovary (CHO) cells, which are the workhorse of recombinant biotherapeutic production, with particular emphasis on antibody products, using strategies such as cell line and protein backbone engineering.

## Introduction

Advances in glycoengineering are increasingly providing opportunities for modulating the properties and functions of recombinant biotherapeutics [1,2]. *In vivo*, glycans are primarily remodelled by targeting glycosylation pathways and the structural environment of a protein surrounding a glycan, whereas *in vitro*, remodelling is achieved by chemoenzymatic methods. *In vivo* strategies will form the focus of this review. Through such studies and using the abundance of data generated in drug development campaigns, we have come to know that the structure and composition of protein-linked oligosaccharides regulates the therapeutic efficacy, immunogenicity and half-life of recombinant biotherapeutics [1]. This is because oligosaccharides can affect glycoprotein conformation in addition to acting as a binding motif and influencing downstream functions such as immune receptor activation. The Quality by Design (QbD) paradigm for the development of recombinant biotherapeutics therefore identifies glycosylation as a critical quality attribute (CQA) [3,4]. Most glycosylated recombinant biotherapeutics are produced in mammalian cell lines because they reproduce human-like glycosylation more closely than bacteria, yeast, plant and insect cell systems. This is key to avoiding undesirable immunogenicity from foreign epitopes. In fact, between 2014 and 2018 the vast majority (84%) of newly approved antibody therapeutics were produced in CHO cells [5]. The ability to design and produce safer, longer lasting and more effective biotherapeutics is extremely valuable to industry and patients alike. However, the non-templated biosynthesis of glycans is a major hurdle that produces heterogeneous glycomic profiles as a result of enzymatic competition for a common substrate, substrate availability and enzyme-substrate specificity [6,7]. This review highlights the importance of N-glycan structure and analytics in biotherapeutic development and aims to illustrate how CHO cell glycoengineering has evolved with increasing speed and precision since the turn of the century.

## Structure-activity relationship of biotherapeutic glycosylation

The role of glycan composition and structure in modulating the effector function of glycoproteins needs to be elucidated in order to determine a priori which glycoform is desirable for a specific biotherapeutic. The intimate structure-activity relationship (SAR) of N-glycans in a therapeutic context is perhaps best studied in monoclonal antibodies (mAbs) [8]. mAbs and their biosimilars

**Box 1. Eukaryotic N-glycosylation**

Mature N-glycans are derived from a complex network of constitutively expressed glycan-remodelling enzymes, which interact with protein-linked oligosaccharides in a sequence of spatially and temporally separated events [12]. Protein N-glycosylation in eukaryotes is initiated in the endoplasmic reticulum (ER) with the formation of a lipid-linked precursor oligosaccharide composed of glucose, N-acetylglucosamine and mannose (abbreviated as Glc_3_Man_9_GlcNAc_2_) which is then translocated to the ER lumen by a flippase [13]. A membrane-bound oligosaccharyl transferase (OST) catalyses the *en bloc* transfer of Glc_3_Man_9_GlcNAc_2_ to the side-chain amino group of an asparagine residue within the conserved protein sequon Asn-X-Ser/Thr (where X is not Pro) [13]. In the ER, glucose residues are trimmed from correctly folded glycoproteins as a signal for further processing in the Golgi apparatus [11]. Glycan remodelling enzymes and nucleotide sugar donor (NSD) transporters are spatially and temporally segregated within the Golgi apparatus [13]. The glycomic profile of a protein is determined by the frequency of stochastic interactions of the glycan and NSD substrates with various glycosyltransferases and glycosidases which form or hydrolyse specific glycosidic linkages between sugar residues. The Golgi network sequentially processes high mannose N-glycans into diverse hybrid and complex structures, with up to four antennae and with or without bisecting β1,4-GlcNAc and other terminal elaborations including sialylation, poly-N-acetyllactosamine (poly-LacNAc) extensions and fucosylation [12]. From the perspective of biotherapeutic production, this complex interplay of reactions achieves poor control of glycan structure. For a more detailed overview of N-glycan biosynthesis please refer to chapter 9 in the Essential of Glycobiology textbook [12]. 
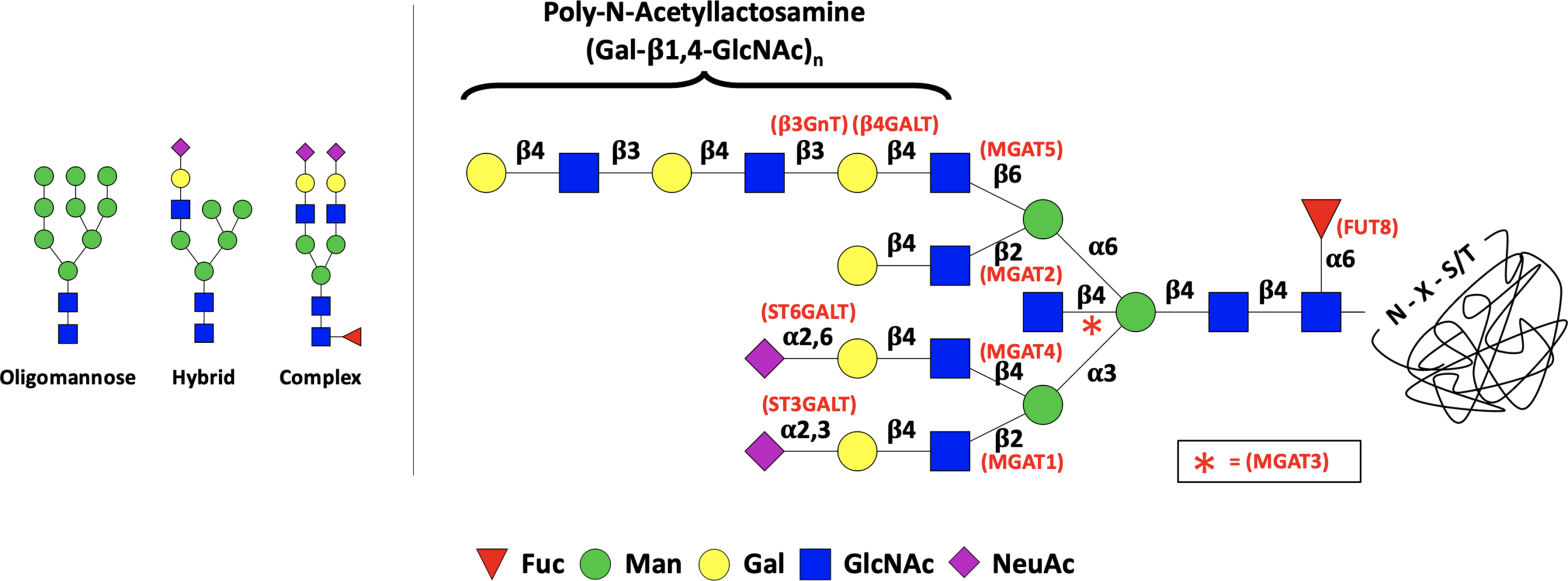

**Figure 1.**
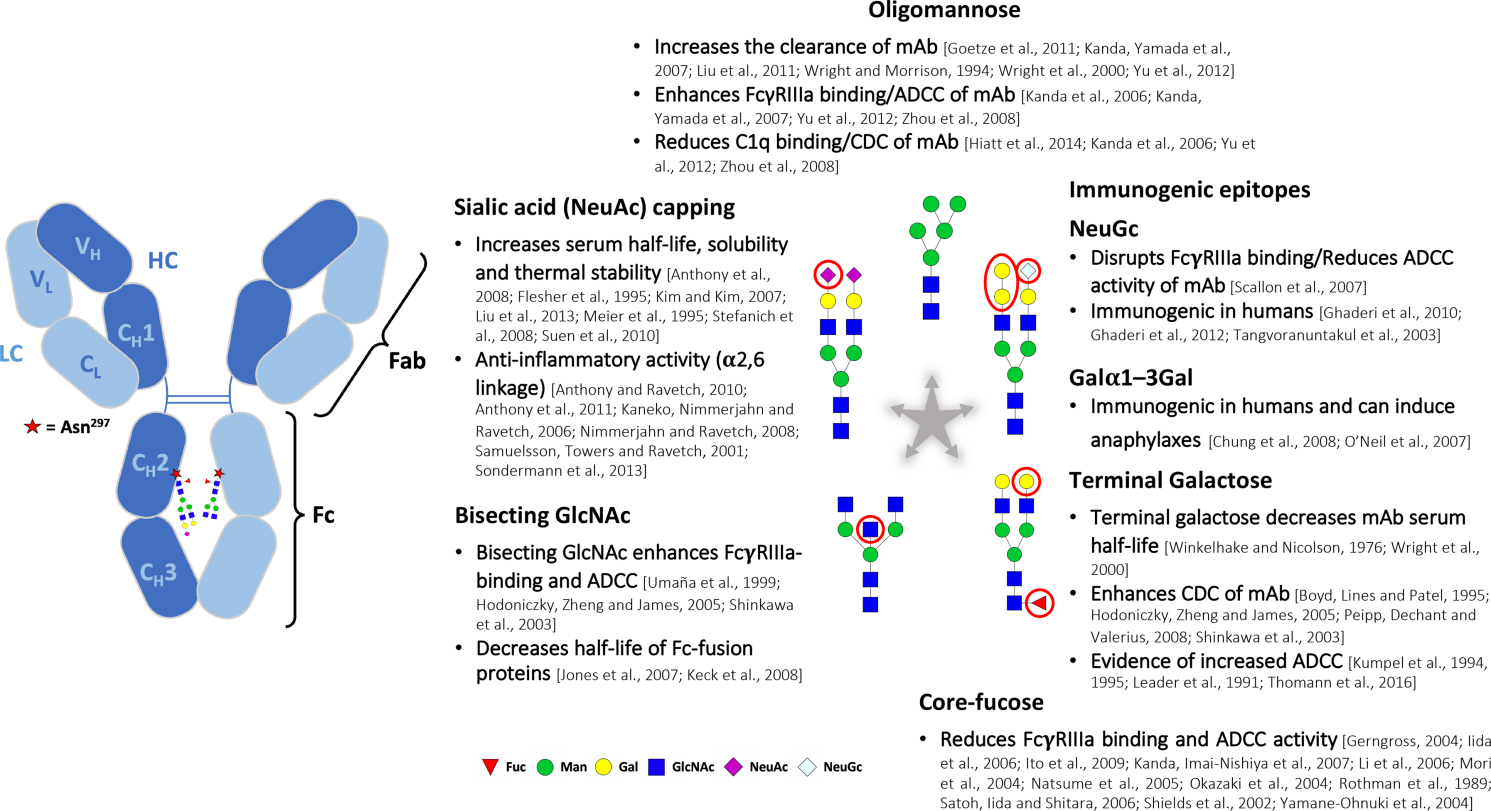
Antibody Fc Glycosylation. IgG requires N-glycosylation at Asn297 in the CH2 domain of the Fc to adopt a functional conformation. The structure-activity relationship of specific Fc-glycosylation characteristics on the pharmacokinetics and pharmacodynamics of mAbs is illustrated. An ensemble of pertinent references is also provided. The hypervariable complementarity determining region in the Fab domain can sometimes contain N-X-S/T sequons with N-glycans whose structure and role is less well understood than in the Fc domain. Different biopharmaceuticals such as hormones, blood-proteins, vaccines, growth factors, interferons and fusion proteins may each require distinct glycomic profiles for optimised function.

On the right-hand side is a schematic structure of a mature mammalian complex N-glycan linked to the conserved N-X-S/T sequon. High mannose glycans from the ER are trimmed sequentially and various terminal elaborations produced in the Golgi are highlighted in this structure. Glycosidic linkages are annotated (in black) together with the Golgi-resident glycosyltransferase genes (in red) responsible for each linkage. Examples of oligomannose, hybrid and complex N-glycans are also shown on the left-hand side.

represent the majority of biopharmaceutical approvals since 2015 and will be the focus of this review [5]. Most therapeutic mAbs are Immunoglobulin G (IgG) which is also the dominant Ig type in human serum. They modulate cellular immune responses through Fcγ receptor (FcγR)-binding as well as through direct receptor- or ligand-binding [9,10]. The conserved Fc site Asn^297^ in IgG1 is N-glycosylated, which is essential to the anti- or proinflammatory role of IgGs (Figure 1) [2]. Disruption of the protein backbone-carbohydrate interface in IgG has been shown to influence the Fc conformations relevant for Fcγ receptor binding [9]. Indeed, without N-glycosylation the Fc portion cannot adopt the functional conformation required to bind Fc-dependent receptors (type 1), glycan dependent receptors (type 2) or initiate the complement cascade [8].

The structure of mAb N-glycans can also influence the pharmacokinetic and pharmacodynamic properties of a biotherapeutic. α1,6 core-linked L-fucose (Fuc) is a primary determinant of IgG inflammatory activity and is added onto N-glycans by an α1,6-fucosyltransferase (FUT8). Core-fucose weakens carbohydrate-carbohydrate interactions necessary for high-affinity binding between antibodies and glycosylated FcγRIIIa, which is why core-fucosylation is undesirable in mAbs that mediate antibody-dependent cell cytotoxicity (ADCC), a mechanism exploited for the selective clearing of tumour cells by immune cells after antibody engagement [14]. The addition of bisecting β1,4-linked N-acetylglucosamine (GlcNAc) by endogenous or recombinant N-acetylglucosaminyltransferase III (GnT-III in YB2/0 and CHO cells respectively) or via *in vitro* glycan remodelling has also been linked to increased ADCC in IgGs, albeit to a lesser extent than afucosylation [15–17]. Antibody fucosylation is reduced by GnT-III overexpression because oligosaccharides with bisecting GlcNAc no longer act as FUT8 substrates [15]. This principle is exploited in the GlycoMab® technology of GlycArt (acquired by Roche) that produces low-fucose biotherapeutics such as the anti-CD20 obinutuzumab, approved in 2013 for treatment of follicular lymphoma and chronic lymphocytic leukaemia, with 10–100-fold improved ADCC [8]. In 2012, mogamulizumab was the first-ever glycoengineered mAb to gain approval and is produced in FUT8 knockout CHO cells (Potelligent® technology) for the treatment of CCR4-positive T cell leukaemia or lymphoma [8]. Enriching the abundance of mAb N-glycoforms with β1,4-linked galactose (Gal) can also be desirable as it has been shown to enhance complement-dependent cytotoxicity (CDC), another mechanism for the selective depletion of tumour cells by Fc activation of the complement pathway [16–21]. In contrast with early studies, enzymatic hypergalactosylation of mAbs was shown to enhance ADCC activity, albeit afucosylation remained the primary influence [22]. One issue with terminally galactosylated mAbs is clearance from circulation by the asialoglycoprotein receptor [8]. On the other hand, galactose is required for sialylation with N-acetylneuraminic acid (NeuAc), which in turn can improve the solubility, anti-inflammatory activity, thermal stability and serum half-life of mAbs [8,23–26]. The type of sialic acid linkage also influences biotherapeutic properties, in fact when NeuAc is α2,3-linked (main sialylated species in CHO-derived glycoproteins) the conformational stability of the C_H_2 domain of IgG can be reduced, most likely a result of a steric effect between the protein backbone and the α2,3-sialylated 6-arm [27,28]. In contrast, it is the α2,6-linked NeuAc (main sialic acid linkage in human IgG) that is solely required for the anti-inflammatory activity of IgG mediated by non-canonical IgG-Fc receptors [24].

Pharmacokinetic studies in both humans and mice revealed that high-mannose structures were cleared more rapidly from circulation, which is likely to be a result of uptake by endogenous receptors such as the mannose receptor [29,30]. While oligomannose glycans (Man_5–9_GlcNAc_2_) have been shown to increase the FcγRIIIa binding and ADCC of mAbs compared with fucosylated complex glycans, the CDC activity was decreased together with the binding affinity to FcγRIIa/b [30]. The lack of core-fucosylation in oligomannose glycoforms will be in part responsible for these properties, highlighting the difficulties in assigning functional properties to a single specific glycan motif. On the other hand, hyper-mannosylation in non-mammalian cell lines such as insect, yeast and plant cells, can be highly immunogenic in humans [8]. N-glycolylneuraminic (sialic) acid (NeuGc), can also be immunogenic in humans, who express a non-functional CMP-N-acetylneuraminic acid hydroxylase and as a result have high-levels of circulating anti-NeuGc antibodies [31,32]. Humans also lack the ability to produce the Galα1–3Galβ1–4GlcNAc–R epitope, which is common in murine cells, and this has been shown to induce anaphylaxis [8,33]. The risks of immunogenic N-glycoforms require a production system that closely mimics human glycosylation, which is one of the reasons why CHO cells are most often used for industrial biotherapeutic production.

## Engineering control of glycan biosynthesis

In glycoengineering, strategies such as cell-line and protein backbone engineering are used effectively to develop production platforms with specific glycosylation abilities and less heterogenous glycomic profiles. CHO cells have become dominant in biotherapeutic production because they produce non-immunogenic and near human-like glycosylation [5]. For instance, CHO cells do not express alpha-1,3-galactosyltransferases, which produce the immunogenic alpha-galactosyl epitope (although they have the gene), and the expression of CMP-N-acetylneuraminic acid hydroxylase, which produces CMP-NeuGc, is virtually absent [27,71,72]. In contrast, while murine cell lines such as NS0 and Sp2/0 also produce glycan structures similar to humans, they also produce immunogenic epitopes such as the Galα(1–3)Gal epitope and they have a high content of NeuGc sialic acid, which is why they are less commonly used for biotherapeutic production [1,73]. For these reasons, CHO cells are the focus of this review and of further discussion of the various strategies aimed at improving biotherapeutics through glycoengineering.

### Cell line engineering

**Figure 2.**
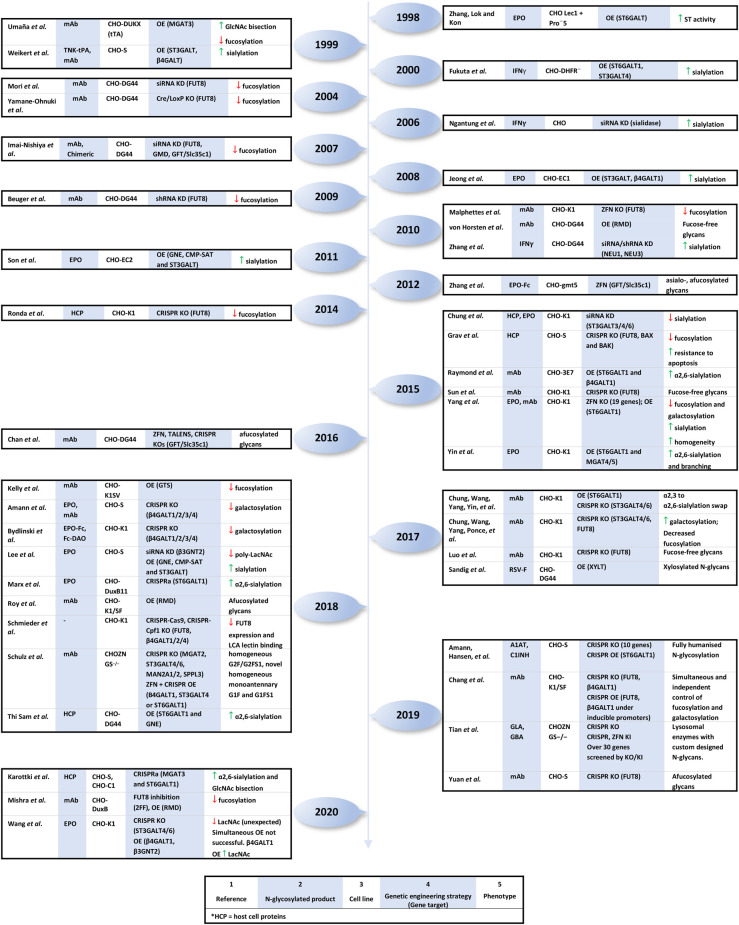
A timeline of genetic engineering in CHO cells for improved control of glycosylation pathways.

In CHO cells, the knock down (KD), knock out (KO) and overexpression (OE) of glycosylation enzymes and NSD transporters have been used in biotherapeutic functional studies and for the development of biobetters, as summarised in Figure 2 [73,74]. The availability of genomic sequences has enabled CHO cell engineering with unprecedented precision using engineered nucleases, including zinc finger nucleases (ZFNs) and transcription activator like nucleases (TALENs) and the clustered regularly interspaced short palindromic repeats (CRISPR) system [71–73,75,76]. These impart the advantage of targeted glycoengineering as opposed to OE using antibiotic selection and, for this reason, are now used as the methods of choice in academic and industrial settings. While mAbs will again be the main focus, relevant glycoengineering strategies in CHO cells applied to other molecules that can inform future work on mAbs will also be discussed. Several studies aiming to enhance sialylation in CHO cells have stably or transiently overexpressed α2,3- and α2,6-sialyltransferases (ST3- and ST6GALTs), which can increase serum half-life and the anti-inflammatory properties of certain mAbs [77–83]. Improved sialyltransferase activity can actually be supplemented with overexpression of branching N-acetylglucosaminyltransferases GnT-IV (*MGAT4*) and GnT-V (*MGAT5*), which add β1,4- and β1,6-linked GlcNAc to the 3- and 6-arms respectively, to increase glycan antennarity and the number of sites available for sialylation [82]. Sialylation can also be indirectly enhanced through increased synthesis of CMP-NeuAc and its transport into the Golgi [84–86]. Since galactose is required for sialylation, overexpression of both β1,4-galactosyltransferase 1 (β4GALT1) and ST3GALT yields even higher sialic acid content compared with ST3GALT overexpression alone [80]. Metabolic engineering has similarly been used in CHO cells to complement the inhibition of poly-LacNAc biosynthesis by removing CMP-Neu5Ac feedback inhibition and to increase sialic acid content in recombinant erythropoietin (EPO) [86,87]. As previously mentioned, afucosylation can be a desirable glycan feature in mAbs designed to generate immune responses, and CHO cells in which GnTIII and FUT8 are overexpressed and knocked out, respectively, have been generated in order to improve ADCC [15,64]. mAb fucosylation has been the primary focus in biopharmaceutical glycoengineering, and just two fucose-engineered products derived from the GlycoMab® and Potelligent® platforms have been approved thus far, although more are undergoing clinical trials. Other strategies aimed at altering the expression levels of glycan remodelling enzymes without affecting the genome often rely on transfection with non-coding RNA such as microRNA (miRNA), short hairpin RNA (shRNA) or small interfering RNA (siRNA) [88,89]. For instance, siRNAs and shRNAs have previously been used to KD neuraminidases in CHO cells to increase sialic acid content of a recombinant human interferon gamma (hIFNγ) [90]. Alternatively, the siRNA-mediated KD of FUT8 in CHO cells has been demonstrated to result in 100-fold improved ADCC for highly afucosylated antibodies [62]. In evaluating the role of glycosylation enzymes, loss of function by complete bi-allelic KO has generally been preferred to partial suppression strategies such as RNA silencing, however the latter is useful in short-term studies [73,91].

The molecular toolbox for CHO cells has recently expanded to take advantage of the CRISPR system [73,75]. A CRISPR-associated (Cas) endonuclease can induce DNA double-strand breaks targeted to a genomic sequence of interest by a programmable guide RNA (gRNA) that forms RNA-DNA base-pairing upstream of an essential trinucleotide protospacer adjacent motif (PAM) [92,93]. This engineering strategy has the advantage that only the gRNA sequence needs to be modified in order to target new genes and the platform is more easily used, and amenable to alternative applications, compared with ZFNs and TALENs [75,76]. Multiple genes can even be targeted with CRISPR in a single ‘multiplexing' step. For instance, FUT8, BAX and BAK (BAX/BAK are pro-apoptotic proteins) were disrupted in CHO cells with the transfection of a single vector containing three single guide RNA (sgRNA - fuses two elements within gRNA) sequences together with Cas9 (from *Streptococcus pyogenes*) to reduce fucosylation and improve resistance to apoptosis [94]. CRISPR-induced KO of target genes can be highly effective since pairs of sgRNAs can be used to excise genes in their entirety, which in turn facilitates the study of gene function without interference from truncated versions of the protein [95–98]. Bydlinski et al. and Amann et al. implemented this in CHO-K1 and CHO-S cells, respectively, to study the influence of individual and stacked galactosyltransferase KOs on N-glycan processing, confirming β4GALT1 as the major contributor to galactosylation in both CHO-K1 and CHO-S and elucidating the minor roles played by β4GALT-2, 3 and 4 [96,99]. A KO of FUT8 has also been generated using CRISPR in CHO cells with the aid of a computational tool ‘CRISPy', for the identification of sgRNA genomic targets as well as potential off-target sequences [100]. In contrast, CRISPR has also been used for the overexpression of transgenes in CHO cells for precise site-specific and efficient genomic integration [101]. CRISPR/Cas9 is now the dominant strategy employed for gene KO and OE in CHO cells because the simple base pairing between sgRNAs and a target genomic site enables more rapid design, ease of use and lower cost compared with the customisable DNA-binding specificities of ZFNs and TALENs.

Glycomic studies of lectin resistant CHO cell mutants and a knock-in/knock-out screening of 19 CHO glycan remodelling enzymes, using EPO and IgG as glycosylation reporters, have provided some of the most comprehensive bases for targeting specific genes to obtain desirable and more homogeneous glycosylation [27,102]. The Yang et al. screen used engineered ZFNs to systematically deconstruct the role of individual glycosylation enzymes, including isoenzymes with overlapping functions [102]. However, a more recent publication by many of the same authors highlighted a shift towards CRISPR-based strategies in CHO cell glycoengineering because it is the most efficient and cost-effective method available, and they have developed a gRNA library to guide future gene targeting efforts [91]. This was put into practice in 2019 when the group performed over 30 KO/KI screens of glycosylation enzymes to produce custom-designed N-glycans for recombinant lysosomal enzymes [103]. Recombinant lysosomal enzymes produced in CHO cells are used to treat lysosomal storage diseases and α-galactosidase A, which is used to treat Fabry disease, was shown to benefit from increased α2,3-sialylation to improve half-life and biodistribution in a mouse model (and lower immunogenicity when pegylated) [103,104]. CRISPR has also been adapted to alternative applications such as CRISPR activation (CRISPRa), where a catalytically inactivated Cas9 was fused to transcription activators to increase the expression of MGAT3 and ST6GAL1 and thus enhance N-glycan bisection and sialylation [105,106]. The CRISPR system has also recently been exploited to generate CHO cells with simultaneously and independently regulated glycosyltransferases under small-molecule inducible promoters [107]. In the future, this particular method could even be adjusted to use temperature or photo-inducible promoters to further expand the repertoire of strategies for glycosylation control. While it is evident from these examples that engineering N-glycosylation in CHO cells is complex and specific to the target biotherapeutic, advances in genetic manipulation strategies are enabling increasingly homogeneous and bespoke glycosylation with unprecedented precision and efficiency.

Although not yet applied in CHO cells, GlycoDelete is an elegant glycoengineering strategy worth mentioning, which reduces glycan heterogeneity by expressing a Golgi-targeted endo-β-N-acetylglucosaminidase in HEK 293SGnTI(−) cells to produce GlcNAc N-glycan ‘stumps’ modified by galactosyltransferases and sialyltransferases to yield small sialylated trisaccharides [108]. The GlycoDelete method has been shown not to affect protein folding or cell physiology and can produce more homogeneous glycoforms ideal for antigen neutralising mAbs with reduced clearance. The last decade has seen the glycoengineering field bloom with new possibilities offered by advances in genetic engineering strategies and increasingly well-characterised production platforms. Combining these strategies with protein backbone engineering promises to further extend our control over biotherapeutic glycosylation.

### Protein backbone engineering

Another major influence on glycosylation is the nature of the recombinant glycoprotein itself. Glycosylation sites can vary both in accessibility and the surrounding amino acid environment on the surface of a protein [109]. These features affect the frequency of interactions with glycan remodelling enzymes and thus generate some control, or limitations on the glycomic profile. Here we provide a brief overview of how glycoengineering can be achieved by targeted modifications to the protein backbone, with a focus on mAb-based products. This is not meant as a comprehensive discussion of all biotherapeutics and cell types, but rather aims to illustrate how cell line engineering can be supplemented in the pursuit of increasingly controlled and homogeneous glycosylation as well as novel glycan functionalities.

IgGs, being some of the most well characterised glycoproteins, have been the subject of structural and mutagenesis studies to identify key residues for receptor-binding [110,111]. Exploiting the structural understanding of glycosylated IgG, further investigation identified residues which profoundly influence glycan processing as a result of protein–carbohydrate interactions. For instance, CHO-expressed IgG1-Fc with four mutations (F241A/F243A/V262E/V264E) has a more open conformation, which increased accessibility and processing by galactosyl- and sialyltransferases [11]. Similarly, mutations altering the protein–carbohydrate interface can also stabilise the C_H_2 domain of IgG and modulate receptor-binding [112]. While these point mutation strategies are highly specific to individual molecules and to distinctive protein–carbohydrate environments, modifications to the protein–carbohydrate interface were also found to be relevant to glycan processing in protein disulfide isomerase, confirming the efficacy and wider applicability of modifying the primary sequence for glycoengineering [113]. Recently, the same group used molecular dynamics simulations to predict glycan accessibility for processing enzymes and confirmed *in vitro* how N-glycan accessibility and tertiary structure modifications affect processing enzyme kinetics at different glycosylation sites in protein disulfide isomerase [114]. The reader is encouraged to read these last two studies to gain a deeper understanding of how protein backbone engineering may be used to direct glycosylation pathways in a broader context.

Efforts aimed at improving control of glycosylation in CHO cells have now allowed an approach which combines genetic engineering of both glycosylation enzymes and the protein backbone. In one study led by the Betenbaugh group, IgG was engineered with the aforementioned mutations to increase glycan processing in CHO cells designed to swap α2,3- for α2,6-sialylation by CRISPR-mediated disruption of two α2,3-sialyltransferases and overexpressing ST6GALT1 [115]. In a second, disruption of ST3GALT4/6 and FUT8 was combined with the same mutations to produce hypergalactosylated IgG for the first time *in vivo* [116].

Alternative applications for backbone modifications are the insertion or removal of novel glycosylation sites into biotherapeutics and the formation of fusion constructs or scaffolds for multimerisation. An early example worth mentioning is the development of darbepoetin alfa, an EPO molecule approved in 2001 with two additional N-oligosaccharides that increased *in vivo* potency and half-life [117]. Coming back to Ig-based biotherapeutics, atezolizumab, an oncolytic anti-PD-L1 mAb, is another clinically approved (2016-US, 2017-EU) glycoengineered product, except the Fc has been engineered to lack glycosylation [5]. With regards to multimerisation scaffolds, polymeric recombinant IgGs have been generated by the insertion of a C-terminal IgM-tailpiece and modifications to Cys residues involved in multimerisation, to generate multimeric forms with increased valency for therapeutic and vaccine applications [118]. The hexameric IgG-Fc fusion, with its additional N-glycosylation site in the IgM-tailpiece, was shown to have increased FcγR binding affinity, which suggested the potential for developing this strategy to replace intravenous immunoglobulin G (IVIG) therapy with a well-defined and more homogeneous biotherapeutic product. This protein-scaffold was later shown to also bind low-affinity receptors (FcRL5, FcγRIIb, and DC-SIGN) with high specificity and avidity [119]. The insertion of a third N-glycosylation site at the N-terminus generated highly branched and hypersialylated glycans, which are mostly absent at the Asn^297^ site and when combined with further mutation of Cys residues, was able to generate a range of monomers and multimers that have tailored interactions with sialic acid-dependent receptors [120]. Some of these hypersialylated mutants were also found to bind influenza viruses with high-affinity and inhibit influenza-mediated hemagglutination [121,122]. While there are several fusion protein approved biotherapeutics, which mostly combine the Fc portion of IgG1 with other proteins, this section aims to highlight how backbone modifications can remove, add or multiply the functional role of glycans through the engineering of glycosylation sites and multimerisation scaffolds [5]. Deconstructing the role of glycans and analysing changes in the glycomic profile of a biotherapeutic is necessary at the developmental stage and essential for quality control purposes and that is why the analytical methods used in support of glycoengineering will be discussed below.

## Glycosylation analysis

Glycomics, the characterisation of released pools of glycans, and glycoproteomics, the characterisation of glycopeptides to give information of the glycans at an individual glycosylation site, are essential in both the glycoengineering field and the biopharmaceutical industry [141]. Firstly, glycosylation analysis is necessary for elucidating the impact of cell line engineering strategies at both the cellular and recombinant product level, and secondly, highly sensitive and reliable analytics are required for strict quality control in the later manufacturing and clinical phases. Combining these methodologies with genomic, transcriptomic, proteomic and metabolomic data has proven to be an effective systems biology strategy for the optimisation of glycosylated biotherapeutics [74]. Secondly, glycosylation is a critical quality attribute of glycoprotein therapeutics. Its role on intended function and molecule stability therefore needs to be evaluated during development and closely monitored during manufacturing as part of process monitoring and quality control protocols.

**Figure 3.**
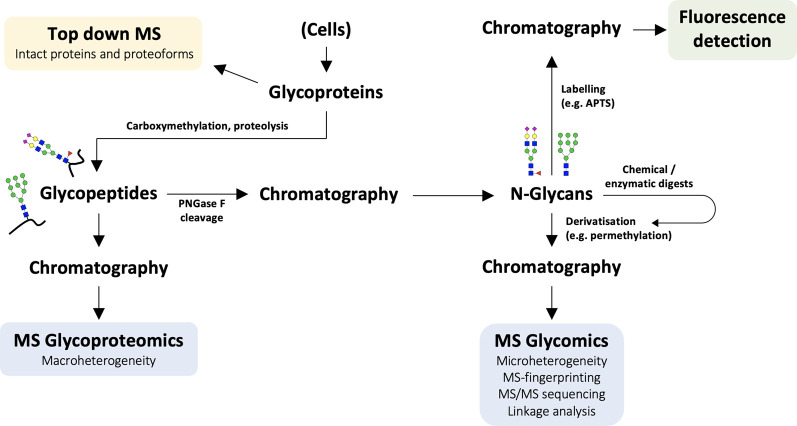
A simplified experimental workflow for N-glycomic or glycoproteomic analyses. Glycoproteins from cells (or purified from supernatant) are reduced/carboxymethylated and proteolytically cleaved. Glycopeptides can then be separated for glycoproteomic analysis or N-glycans can be released by peptide N-glycosidase F (PNGase F). After purification, N-glycans can either be labelled for fluorescence detection or derivatised (e.g. permethylated) to improve ionisation efficiency and sensitivity for MS glycomic analysis. Additionally, MS/MS sequencing or chemical/enzymatic digests can aid more in-depth structural analyses of linkages, antennarity and terminal epitopes.

Mass spectrometry (MS)-based techniques, the experimental workflow for which is outlined in Figure 3, have dominated the glycomics and glycoproteomics fields for decades because of its high sensitivity and ability to characterise complex heterogeneous mixtures, especially when combined with upstream separation techniques such as liquid chromatography [142]. Chromatographic techniques such as hydrophilic interaction chromatography (HILIC) and capillary electrophoresis continue to improve and have also been used together with fluorescence detection of glycans to obtain quantitative glycomic profiles, however these methods are hindered in their ability to assign glycan structures to specific signals [143,144]. In comparison with fluorescence detection data, the m/z (mass/charge) molecular ion signals obtained via MS are more easily assigned to glycan structures using prior knowledge of N-glycan biosynthesis during mass-fingerprinting, because a given mass will usually have a unique monosaccharide composition. While the 1-dimensional MS mode can typically determine monosaccharide composition unambiguously, structural isomers with varied branching patterns or epitopes cannot be distinguished. For more detailed structural elucidation, two-dimensional tandem-MS/MS uses collision-induced dissociation to produce sequence informative fragment ions. Additionally, enzymatic digestions of specific glycan residues can provide another level of structural detail. Matrix-assisted laser desorption/ionisation (MALDI) and electrospray ionisation (ESI) are ‘soft ionisation' techniques, which now dominate in the MS field [142,145]. MALDI in particular has risen to prominence for its exquisite sensitivity, tolerance to impurities, high-throughput capacity, ease of data analysis (given that most ions are singly charged) and the simplicity of sample preparation [146]. More comprehensive reviews of these methodologies are provided (an overview is also provided in Table 1) [142,145–149].

**Table 1:** Chromatographic and MS techniques for glycomic analyses

Separation	Description	Advantages	Disadvantages
CE	Capillary electrophoresis	High separation efficiency and miniaturisation capacity. Used for separation of native or labelled glycans	Low flow rate not suited to MS. Typically associated with laser-induced fluorescence detection, which complicates peak annotation. More suited to less heterogeneous samples (mAb Fc N-glycans)
PGC	Porous graphitised carbon chromatography	A widely used method for purifying and separating underivatised glycans. Resolves structural isomers efficiently	Limited robustness and reproducibility.
RPLC	Reverse phase liquid chromatography	C18 is commonly used to elute and purify released N-glycans while peptides are retained. Permethylated glycans are retained and separated well. Highly reproducible	Does not retain hydrophilic glycans or more hydrophilic glycopeptides. Not efficient for isomer separation
HILIC	Hydrophilic interaction liquid chromatography	Efficient separation of underivatised glycans	Less effective than RPLC for hydrophobic molecules and PGC for isomer separation
GC	Gas chromatography	Suited to electron impact ionisation and linkage analysis	Requires volatile sample. Limited to lower Mw molecules
Ion mobility	Molecules are separated by size and shape (in addition to mass/charge) after ionisation by drifting through an inert gas	Can resolve compositional, conformational and linkage isomers	Larger and highly similar glycans separate less efficiently. Sample must be in gas phase
Immunoaffinity	Immunoprecipitation using antibodies	Highly specific separation of glycan epitopes	For each antibody only a single glycan epitope can be purified
Lectin affinity	Lectin affinity chromatography	Highly specific separation of glycan epitopes	For each lectin only a single glycan epitope can be purified
**Ionisation**			
EI	Electron impact ionisation. Gas phase molecules are bombarded by a beam of electrons	Non-selective, efficient ionisation can result in high sensitivity. Can be used in GC workflow for linkage analysis.	Requires volatile (gaseous) sample, less suited to polymers. Only forms positive ions. Hard ionisation can cause unwanted fragmentation. Not suited to liquid chromatography
ESI	Electrospray ionisation. High voltage applied on a flow of liquid at atmospheric pressure	Can ionise high Mw samples in both positive and negative mode. Utilised for top-down analyses. Perfectly suited to liquid chromatography integration.	Sample must be in solution. Complicated spectra as multiply charged ions are produced
MALDI	Matrix assisted laser/desorption ionisation. Laser pulse is applied to a sample embedded in a matrix	Ionises large molecules. Excellent resolving power and sensitivity for highly heterogeneous mixtures. Can be coupled to offline chromatography.	Sample must be embedded in a solid matrix. Ionisation suppression possible
**Mass analysers**			
Quadrupole	Ions of specific m/z selected with radiofrequency and direct current voltages	Low cost and fast. Useful for m/z filtering prior to second analyser	Lower resolution and limited mass range
TOF	Time-of-flight. Larger m/z ions travel slower than smaller m/z ions. Reflectrons increase path length and sensitivity	High resolving power, fast, sensitive, and theoretically unlimited mass range	Lower resolving power than Orbitrap and FT-ICR
Orbitrap	Ions are electrostatically trapped in orbit around a central spindle. Oscillation frequency depends on m/z	Very high resolving power and mass range	High cost
FT-ICR	Fourier transform ion cyclotron resonance. The frequency of cyclotron motion caused by the ions trapped in a magnetic field is measured. Motion frequency is dependent on ion m/z	Extremely high resolving power and sensitivity. Amenable to high m/z ranges	Relatively slow speed. High initial costs and maintenance of superconducting magnet

## Conclusions

This review has examined the functional role of biotherapeutic glycosylation, strategies for CHO cell line and protein backbone engineering aimed at glycosylation control and the analytical methods commonly used for glycomic and glycoproteomic analyses. Advances in various omics technologies have significantly improved our understanding of CHO cells at different levels and enabled the rational targeting of glycosylation effectors for improved biopharmaceutical production [27,71,72,150–152]. A remarkable and growing list of studies is revolutionising our understanding of the fundamentals of protein glycosylation and advances in genome editing have made glycoengineering increasingly accessible and efficient for the development of biobetters with improved half-life, safety and efficacy. Despite a focus on increasing viable cell density and specific productivity from industry, we expect that glycoengineering will form an increasingly intrinsic part of biopharmaceutical design and development.

## Perspectives

- Glycosylation is a critical quality attribute that influences the efficacy, half-life and safety of recombinant biotherapeutics. However, control of this pathway is made difficult due to the non-template-based synthesis of glycans.
- Over the last two decades, increasingly effective genetic engineering strategies have enabled cell line development and protein backbone engineering with unprecedented precision and speed. Modelling work, optimisation of process conditions and *in vitro* strategies are also being pursued in parallel.
- The effects of glycoengineering on cell physiology and N-glycosylation in contexts other than IgG-Fc and EPO will need to be further investigated. Host cell proteins, ‘Glycan scaffolds', glycosylated Fab domains and other recombinant products are of significant interest.

## Open Access

Open access for this article was enabled by the participation of Imperial College London in an all-inclusive *Read & Publish* pilot with Portland Press and the Biochemical Society under a transformative agreement with JISC.

## Abbreviations

ADCC: antibody-dependent cell cytotoxicity
CDC: complement-dependent cytotoxicity
CHO: Chinese hamster ovary
CRISPR: clustered regularly interspaced short palindromic repeats
EPO: erythropoietin
ER: endoplasmic reticulum
ESI: electrospray ionisation
FUT8: α1,6-fucosyltransferase
HILIC: hydrophilic interaction chromatography
KD: knock down
KO: knock out
MALDI: Matrix-assisted laser desorption/ionisation
MS: mass spectrometry
NSD: nucleotide sugar donor
OE: overexpression
TALENs: transcription activator like nucleases
ZFNs: zinc finger nucleases
